# Berberine Induces Caspase-Independent Cell Death in Colon Tumor Cells through Activation of Apoptosis-Inducing Factor

**DOI:** 10.1371/journal.pone.0036418

**Published:** 2012-05-04

**Authors:** Lihong Wang, Liping Liu, Yan Shi, Hanwei Cao, Rupesh Chaturvedi, M. Wade Calcutt, Tianhui Hu, Xiubao Ren, Keith T. Wilson, D. Brent Polk, Fang Yan

**Affiliations:** 1 Department of Pediatrics, Vanderbilt University Medical Center and the Monroe Carell Jr. Children’s Hospital at Vanderbilt, Nashville, Tennessee, United States of America; 2 Cancer Research Center, Xiamen University Medical College, Xiamen, P. R. China; 3 Department of Medicine, Vanderbilt University Medical Center, Nashville, Tennessee, United States of America; 4 Department of Biochemistry, Vanderbilt University Medical Center, Nashville, Tennessee, United States of America; 5 Department of Biotherapy, Cancer Institute & Hospital, Tianjin Medical University, Tianjin, P. R. China; 6 Department of Cancer Biology, Vanderbilt University Medical Center, Nashville, Tennessee, United States of America; 7 Veterans Affairs Tennessee Valley Healthcare System, Nashville, Tennessee, United States of America; 8 Department of Pediatrics, University of Southern California and Children’s Hospital Los Angeles, Los Angeles, California, United States of America; Universidade Federal do Rio de Janeiro, Brazil

## Abstract

Berberine, an isoquinoline alkaloid derived from plants, is a traditional medicine for treating bacterial diarrhea and intestinal parasite infections. Although berberine has recently been shown to suppress growth of several tumor cell lines, information regarding the effect of berberine on colon tumor growth is limited. Here, we investigated the mechanisms underlying the effects of berberine on regulating the fate of colon tumor cells, specifically the mouse immorto-Min colonic epithelial (IMCE) cells carrying the *Apc* ^min^ mutation, and of normal colon epithelial cells, namely young adult mouse colonic epithelium (YAMC) cells. Berberine decreased colon tumor colony formation in agar, and induced cell death and LDH release in a time- and concentration-dependent manner in IMCE cells. In contrast, YAMC cells were not sensitive to berberine-induced cell death. Berberine did not stimulate caspase activation, and PARP cleavage and berberine-induced cell death were not affected by a caspase inhibitor in IMCE cells. Rather, berberine stimulated a caspase-independent cell death mediator, apoptosis-inducing factor (AIF) release from mitochondria and nuclear translocation in a ROS production-dependent manner. Amelioration of berberine-stimulated ROS production or suppression of AIF expression blocked berberine-induced cell death and LDH release in IMCE cells. Furthermore, two targets of ROS production in cells, cathepsin B release from lysosomes and PARP activation were induced by berberine. Blockage of either of these pathways decreased berberine-induced AIF activation and cell death in IMCE cells. Thus, berberine-stimulated ROS production leads to cathepsin B release and PARP activation-dependent AIF activation, resulting in caspase-independent cell death in colon tumor cells. Notably, normal colon epithelial cells are less susceptible to berberine-induced cell death, which suggests the specific inhibitory effects of berberine on colon tumor cell growth.

## Introduction

Berberine is an isoquinoline alkaloid isolated from several plants, such as *Hydrastis canadensis* (goldenseal), *Berberis aquifolium* (oregon grape), and *Berberis vulgaris* (barberry). Berberine has been used to treat bacteria-associated diarrhea, intestinal parasitic infections, and ocular trachoma infections for several decades through its antimicrobial activities [1]. Various beneficial effects of berberine on several diseases have also been reported. For example, berberine’s immunoregulatory potential has been demonstrated by inhibiting HIV protease inhibitor-induced TNF and IL-6 production in macrophages [2], enhancing progression of type 1 diabetes in mice, and decreasing Th17 and Th1 cell differentiation and cytokine production [3]. Other effects of berberine on diseases include reducing cholesterol levels in humans and hamsters by elevating LDL receptor expression [4], inhibiting hepatic gluconeogenesis to improve glucose metabolism in diabetic rats [5], and reducing the permeability of the blood-brain barrier and attenuating autoimmune encephalomyelitis in mice [6].

Recently, increasing evidence supports the inhibitory effect of berberine on growth of broad tumor cell types derived from bone marrow, liver, lung, gastrointestinal tract, oral, skin, brain, bone, bladder, breast, cervix, and prostate [7], [8]. Several mechanisms have been reported for berberine’s antitumor activity. Berberine has been shown to suppress cancer cell growth and proliferation by inducing cell cycle arrest, stimulate cancer cell caspase-dependent apoptosis, reduce Bcl-2 and Bcl-xL levels and increase Bax and Bak levels, and inhibit metastasis by downregulating matrix metalloproteinases. Signaling pathways involved in anti-cancer effects of berberine include p53, MAPK, and NF-κB [7], [8]. These findings indicate the multiple mechanisms involved in anti-cancer effects of berberine on different tumor cell types.

Apoptosis, occurring in a caspase-dependent manner, is the best-known modality of programmed cell death. Two canonical pathways have been shown to regulate caspase-dependent apoptosis, extrinsic “death-receptor-mediated” and intrinsic “mitochondrial-mediated” [9]. The extrinsic pathway includes recruitment of adaptor molecules that activate caspase-2, -8 or -10. For the intrinsic pathway, mitochondrial outer membrane permeabilization causes cytochrome -c release, which binds caspase-9 to assemble a cytoplasmic complex called the apoptosome. These two pathways converge in the activation of caspase-3 and/or caspase-6 and -7. Both extrinsic and intrinsic apoptotic pathways are associated with the activation of caspase-activated DNase, which generates nuclear oligonucleosomal DNA fragmentation [9], [10].

Programmed cell death can also occur through an alternative mitochondrial route, which is independent of caspase activation [11]. In this case, loss of mitochondrial function results in release of mitochondrial proteins to induce cell death without activation of caspases. Apoptosis-inducing factor (AIF), a mitochondrial oxidoreductase, is one of the best-studied mediators stimulating caspase-independent cell death [12], [13]. AIF localizes normally in the intermembrane space of mitochondria. Upon cellular insult, AIF is cleaved by activated poly (ADP-ribose) polymerase (PARP)-1, and/or two cysteine proteases, calpains and cathepsins to yield truncated AIF (tAIF) [14]. tAIF relocates from the mitochondria to the cytosol and the nucleus, where it plays a key role in provoking large-scale DNA degradation and chromatin condensation [13]. Oxidative damage, such as generation of reactive oxygen species (ROS), has been shown to mediate PARP-1 activation and lysosomal permeabilization, triggering cathepsin B release, mitochondrial dysfunction, and AIF release, which leads to caspase-independent cell death [15], [16].

Colon cancer is one of the leading causes of cancer death in the developed world. Since the information regarding berberine’s effects on colon cancer development is limited, the purpose of this work was to investigate the mechanisms of action of berberine in colon tumor cells. Here, we report that berberine induces ROS-mediated stimulation of AIF activation through cathepsin B release and PARP activation, which leads to caspase-independent cell death in colon tumor cells. However, normal colon epithelial cells are not as sensitive to berberine-induced cell death as colon tumor cells. These findings suggest that berberine may provide relative selectivity for colon cancer therapy, with less cytotoxic effects on normal colon epithelial cells.

## Results

### Berberine Induces Cell Death and LDH Release in Colon Tumor Cells

Berberine has been shown to suppress cell growth in tumor cell lines through various mechanisms [7], [8]. However, information regarding the effect of berberine on colon cancer development is limited. Thus, we determined the effects of berberine on colon tumor cells. Familial adenomatous polyposis (FAP) can be caused by germline mutations in the adenomatous polyposis coli (*Apc*) tumor suppressor gene. The multiple intestinal neoplasia (Min) mouse has a germline mutation in the *Apc* gene and develops multiple polyps in the intestine. Thus, the *Apc* ^min/+^ mouse is an animal model of human FAP [17]. We used mouse immorto-Min colonic epithelium (IMCE) cells isolated from the colonic epithelium of the *Apc* ^min/+^ mouse crossed with the Immortomouse as a colon cancer cell model. Another colon tumor cell line, HT-29 cells, a human colon carcinoma cell line, was also studied. The young adult mouse colonic epithelial (YAMC) cells were used to assess berberine’s effects on normal colon epithelial cells.

**Figure 1:**
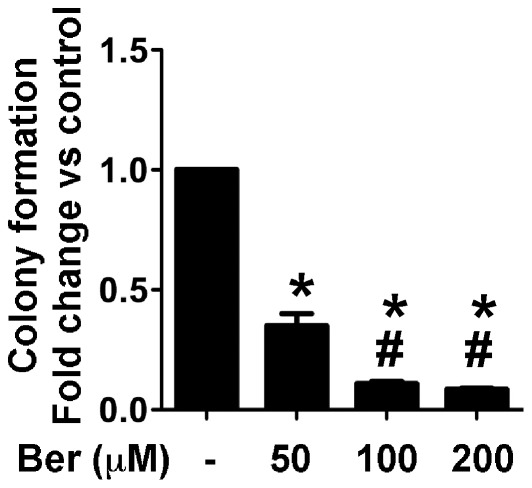
Berberine inhibits colon tumor colony growth in soft agar. IMCE*^ras^* cells were cultured in 0.35% agar on the basal 0.5% agar’s layer in IMCE cell culture medium in the presence or absence of the indicated concentrations of berberine at 33°C for 14 days. Prior to counting colonies, cells were stained using the CellTiter®120 AQueousOne Solution Cell Proliferation Assay. Colonies of >50 µm were counted. The data are shown as fold increase in comparison with the colony cultured without berberine (control). Data represent at least 3 independent experiments, each performed in triplicate. *p<0.01 compared to the control group, #*p*<0.01 compared to the 50 µM of the berberine treated group.

We first detected the effects of berberine on tumor cell colony formation. Overexpression the oncogene, *ras*, in IMCE (IMCE*^ras^* ) cells promotes formation of colonies in agar. Co-culture of berberine with IMCE*^ras^* cells markedly decreased the colony counts (Figure 1). These results indicate that berberine is capable of inhibiting colon cancer cell growth.

**Figure 2:**
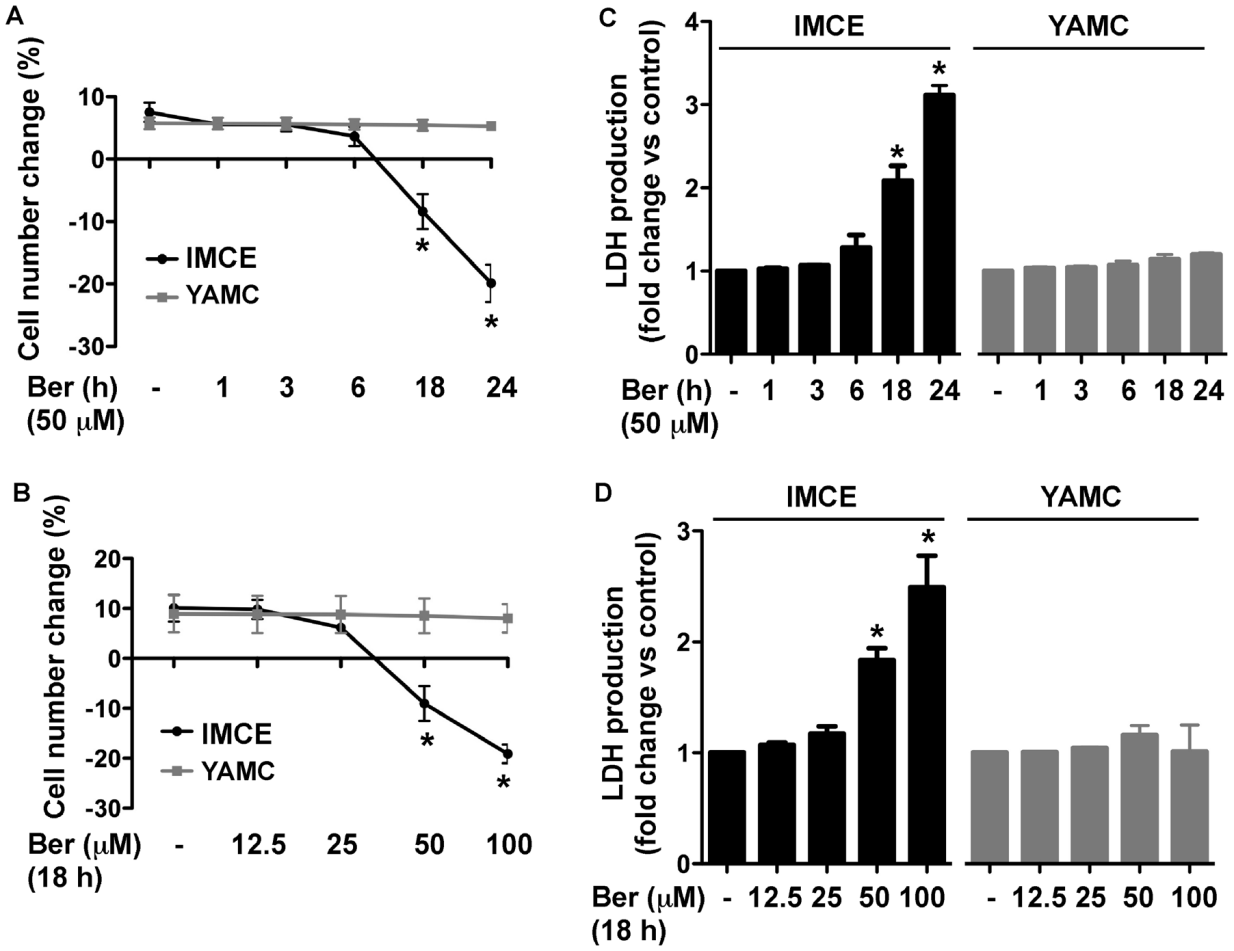
Berberine induces cell death and LDH release in the colon cancer cell line, IMCE cells. IMCE and YAMC cells were cultured in serum-starved RPMI 1640 medium at 37°C (non-permissive condition) for 24 h with or without 50 µM berberine treatment for indicated times from the end of experiment (A and C) or berberine treatment at indicated concentrations for 18 h (B and D). Cell viability was assessed using the CellTiter®729 AQueousOne Solution Cell Proliferation Assay (A–B). % of cell number change was calculated as: [(cell number at the end of treatment-cell number before treatment)/(cell number before treatment)]×100. Cell culture supernatants were collected for detecting LDH release using the CytoTox 96® 159 Non-Radioactive Cytotoxicity Assay (C–D). LDH release is expressed as a percentage of control in each experiment. Data represent at least 3 independent experiments, each performed in triplicate. *p<0.01 compared to the control group.

To further determine the potential role of berberine in regulating the fate of colon cell lines, we treated IMCE, HT-29, and YAMC epithelial cells with berberine at 50 µM for 1, 3, 6, 18, and 24 h and then analyzed cell viability. Berberine treatment for 18 h and 24 h resulted in a significant reduction of cell number in IMCE cells (Figure 2A). When cells were treated with berberine at 12.5, 25, 50, 100 µM for 18 h, berberine treatment at 50 µM and 100 µM resulted in a significant induction of the death of IMCE cell lines (Figure 2B).

The release of intracellular components, such as LDH, is a marker for cell death. We therefore assessed whether treatment of cells with berberine resulted in LDH release. Berberine stimulated LDH release from IMCE cells in a time- and concentration-dependent manner (Figure 2C–D).

In addition to IMEC cells, berberine induced cell death (Figure S1A) and LDH release (Figure S1B) in HT-29 cells in a concentration-dependent manner. Furthermore, berberine showed significant increased effects on cell death when cells were treated with berberine for 36 h, compared to 24 h treatment (Figure S1C and S1D).

Notably, in YAMC cells, berberine did not induce cell death (Figure 2A–B) or LDH release (Figure 2C–D) at 50 µM and 100 µM with 18 h or 24 h treatment. No loss of YAMC cells detected using the cell viability assay was found when cells were treated with berberine at concentrations from150 µM to 300 µM (Figure S2A). Berberine at concentrations of higher than 150 µM increased LDH release in YAMC cells (Figure S2B). The effects of 200 µM of berberine treatment on LDH release in YAMC cells was observed at the similar levels as those induced by 50 µM of berberine treatment in IMCE cells. These data suggested that colon tumor cells are more sensitive to cell death by berberine treatment, compared to normal colon epithelial cells.

### Berberine-induced Cell Death is Caspase-independent in IMCE Cells

**Figure 3:**
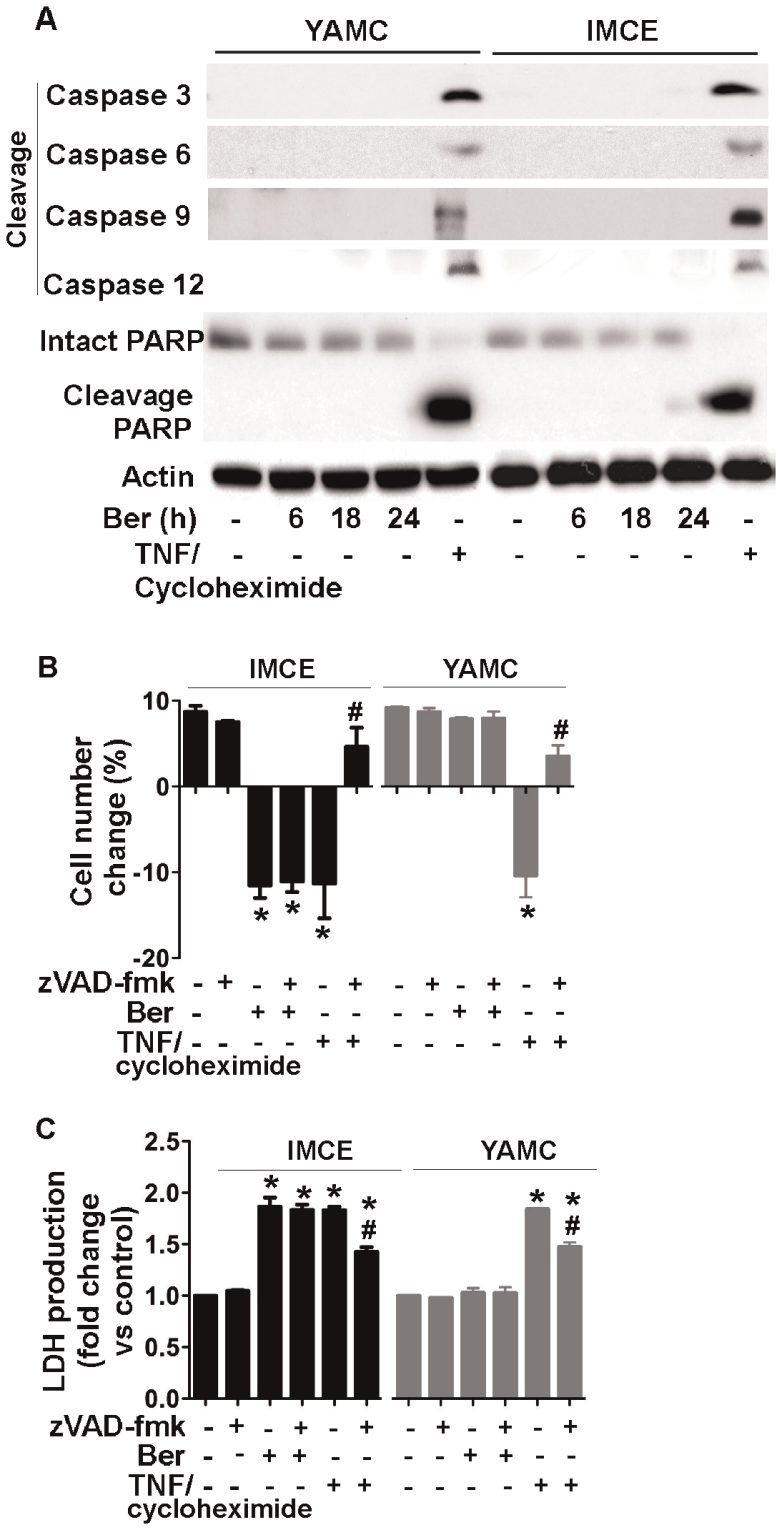
Berberine induces cell death through a caspase-independent mechanism in IMCE cells. Cells were treated with berberine at 50 µM for the indicated times or TNF (100 ng/ml) and cycloheximide (1 µg/ml) for 6 h in serum-starved RPMI 1640 medium at 37°C, as described in Figure 2. Cellular lysates were collected for Western blot analysis using antibodies against cleavage (active) forms of caspases and an anti-PARP antibody (which identifies both intact and cleavage forms) (A). The actin blot was used as a protein loading control. Cells were treated with berberine at 50 µM for 18 h or TNF and cycloheximide for 6 h in the presence or absence of a caspase inhibitor, zVAD-fmk (25 µM). Cell number change (B) and LDH production (C) were detected as described in Figure 2. *p<0.01 compared to the control group. #*p*<0.01 compared to the TNF/cycloheximide treated group.

Since caspase-dependent apoptosis is the best-known modality of programmed cell death, we determined whether berberine-induced cell death is due to apoptosis in IMCE cells. We analyzed caspase activation and PARP cleavage in cells treated with berberine. None of the caspases, including caspase-3, caspase-6, caspase-9, and caspase-12 were activated by berberine in IMCE and YAMC cells (Figure 3A). In addition, berberine did not stimulate cleavage of PARP (Figure 3A) in these two cell lines. Cells were treated with TNF and cycloheximide as a positive control for stimulating apoptosis. TNF and cycloheximide cotreatment activated caspases-3, 6, 9 and 12 and stimulated PARP cleavage in both IMCE and YAMC cells (Figure 3A). Consistent with these data, berberine-stimulated cell death and LDH release in IMCE cells were not blocked by the caspase inhibitor, zVAD-fmk, in IMCE cells (Figure 3B–C). However, zVAD-fmk inhibited TNF and cycloheximide-induced cell death in both IMCE and YAMC cells (Figure 3B–C).

Berberine-induced cell death was also detected using Annexin V and propidium iodide (PI) staining and flow cytometry. The cell population with Annexin V positive and PI negative represents cells in early apoptosis. The cell population with Annexin V positive and PI positive is found in late apoptosis and in AIF-mediated caspase-independent program cell death [18]. Positive Annexin V staining never appears before permeabilization of the plasma membrane (assessed by PI staining). Thus, there is no Annexin V positive and PI negative cell population in caspase-independent cell death. In agreement with these published results, we found that berberine increased the cell population with Annexin V positive and PI positive, but not the cell population with Annexin V positive and PI negative (Figure S3). These data indicate that berberine induced colon tumor cell death in a caspase-independent manner.

### Berberine-induced Caspase-independent Cell Death in IMCE Cells Requires ROS Production

**Figure 4:**
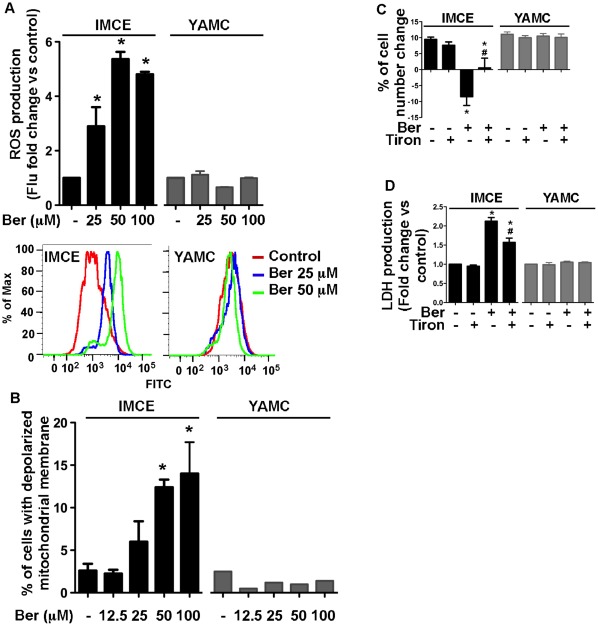
Berberine-induced ROS production is required for cell death in IMCE. Cells were treated with berberine at indicated concentrations for 18 h (A and B), as described in Figure 2. Intracellular ROS levels were detected using DHE probe and the amount of fluorescence was measured using flow cytometry. The fold change of the amount of fluorescence was calculated by comparing that in the treated groups to the control group (A). Cells were treated with the JC-1 probe and then analyzed by flow cytometry to measures the fraction of cells with polarized or depolarized mitochondrial membrane (B). Cells were treated with berberine at 50 µM for 18 h in the presence or absence of 5 µM of Tiron, a ROS scavenger. Cell number change (C) and LDH release (D) were detected as described in Figure 2. *p<0.01 compared to the control group, #*p*<0.01 compared to the berberine treated group.

Berberine has been shown to stimulate ROS production in several types of tumor cells to induce apoptosis [19], [20]. However, other studies reported that generation of ROS mediates caspase-independent cell death [15], [16]. Here, we determined whether berberine-induced ROS production served as a possible upstream mediator of caspase-independent cell death in IMCE cells. ROS production was detected using dihydroethidium, a cell-permeable fluorescence dye that reacts with a broad spectrum of ROS and is analyzed by flow cytometry. The results showed that berberine treatment resulted in an increase in ROS production in a concentration-dependent manner only in IMCE cells, but not in YAMC cells (Figure 4A). In addition to ROS production, we measured mitochondrial membrane potential by staining cells with JC1 dye. We found that berberine induced mitochondrial depolarization in a concentration-dependent manner in IMCE cells, but not in YAMC cells (Figure 4B).

We further detected the effects of berberine-induced ROS production on cell death in IMEC cells. Tiron, a cell permeable ROS scavenger, significantly down-regulated berberine-inducted ROS level (Figure S4). Pretreatment of cells with Tiron decreased cell death and release of LDH in IMCE cells treated with berberine (Figure 4C–D). These data suggest that ROS generation by berberine is required for colon tumor cell death. This conclusion is further supported by the fact that no ROS production and cell death (Figure 2) was observed in YAMC cells treated with berberine.

### Berberine Induces AIF Release from the Mitochondria and Nuclear Translocation, Which is Required for Cell Death in IMCE

**Figure 5:**
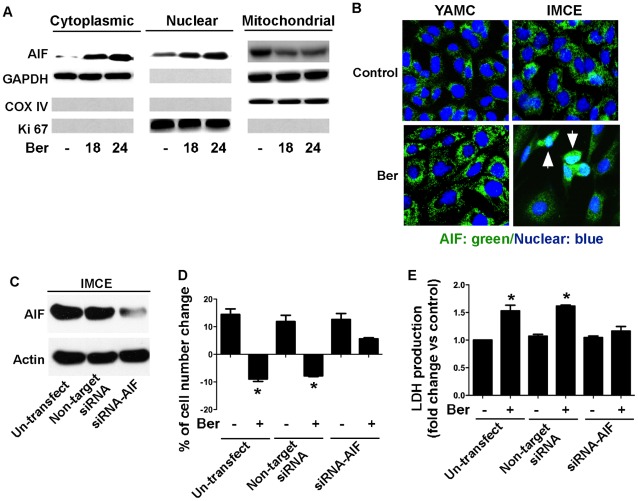
Berberine stimulates AIF release from the mitochondria to the cytoplasm and nuclear translocation in IMCE, which is required for berberine-induced cell death. Cells were treated with berberine at 50 µM for the indicated times, as described in Figure 2. Cytoplasmic, nuclear, and mitochondrial fractions were prepared for Western blot analysis using anti-AIF, anti-GAPDH (cytosolic marker), anti-COX IV (mitochondrial marker) and anti-Ki-67 (nuclear marker) antibodies (A). Immunostaining of cells with 50 µM berberine treatment for 18 h was performed to localize AIF using an anti-AIF antibody and a FITC-conjugated secondary antibody (green) and nuclei using DAPI staining (blue). Arrows indicate nuclei with AIF translocation (B). IMCE cells transfected with AIF siRNA or non-targeting siRNA for 24 h were treated with berberine at 50 µM for 18 h to detect number change (D) and LDH release (E) as described in Figure 2. AIF expression levels were detected by Western blot analysis using an anti-AIF antibody (C). Actin blot was used as a protein loading control. *p<0.01 compared to the control group.

As a consequence of ROS production, loss of mitochondrial membrane potential may result in caspase-independent cell death. To determine whether the mitochondrial pathway was involved in induction of cell death by berberine, we examined the effect of berberine on AIF activation. AIF is a mediator known to be involved in the induction of the caspase-independent pathway. AIF is released from mitochondria in response to death stimuli, and subsequently, translocated into the nucleus leading to nuclear condensation. We analyzed AIF localization by Western blotting analysis of fractioned cellular components. Berberine treatment induced AIF release to the cytosol and translocation to the nuclei because AIF accumulated in the cytosolic and nuclear fractions, and less AIF was found in the mitochondria (Figure 5A). Moreover, immunofluorescence showed a granular pattern (mitochondrial distribution) in control, whereas AIF was detected in the nuclei upon berberine treatment in IMCE cells, but not in YAMC cells (Figure 5B). Berberine-induced AIF activation was also detected in HT-29 cells (Figure S5A).

To further study the role of AIF activation in berberine-induced cell death, we knocked down AIF expression in IMCE cells by transfecting AIF targeted siRNA (Figure 5C). We found that down-regulation of AIF expression ameliorated cell death and LDH release induced by berberine in IMCE cells, compared to non-transfected cells or cells transfected with a non-targeting siRNA (Figure 5D–E). These results indicate the requirement of AIF release from the mitochondrial to cytoplasmic for berberine-induced cell death in tumor cell lines.

### Cathepsin B Release Mediates Berberine-stimulated AIF Activation and Cell Death in IMCE Cells

**Figure 6:**
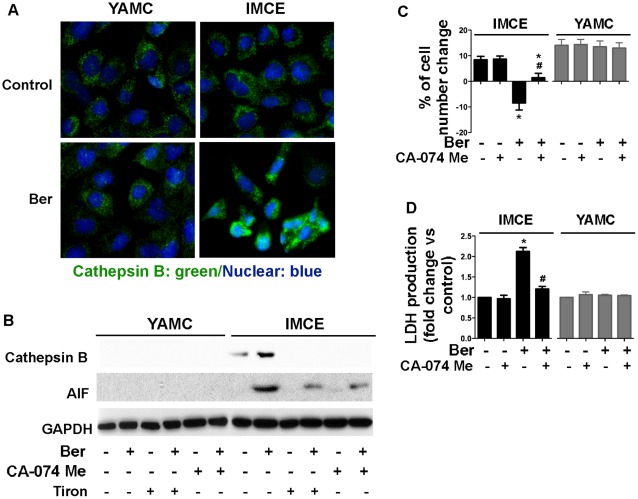
Berberine-stimulated cathepsin B release through ROS production is required for AIF activation in IMCE. Cells were treated with berberine at 50 µM for 18 h in the presence or absence of a ROS scavenger, Tiron (5 µM) or a cathepsin B inhibitor, CA-074 Me (100 nM). Immunostaining was performed to localize AIF using an anti-cathepsin B antibody and a FITC-conjugated secondary antibody (green) and nuclei using DAPI staining (blue) (A). Cathepsin B staining shows granular lysosomal pattern in control cells and berberine treated YAMC cells and a diffuse cytosolic pattern with more nuclear distribution after berberine treatment in IMCE cells. Cytoplasmic fractions were prepared for Western blot analysis using anti-cathepsin B, anti-AIF, anti-GAPDH antibodies (B). Cell number change (C) and LDH release (D) were detected as described in Figure 2. *p<0.01 compared to the control group, #*p*<0.01 compared to the berberine treated group.

Since several studies have reported that ROS production induced permeabilization of lysosomes, we tested whether berberine-induced ROS production could stimulate cathepsin-B release. Immunofluorescence results confirmed that berberine induced cathepsin-B release from the lysosome and nuclear translocation in berberine-treated IMCE cells, but not YAMC cells (Figure 6A). To investigate the role of ROS in AIF activation, possibly through cathepsin B release from lysosomes, we pretreated IMCE and YAMC cells with the ROS scavenger, Tiron, or the cathepsin B inhibitor CA-074 Me. Tiron inhibited berberine-induced cathepsin-B and AIF release into the cytosol (Figure 6B). CA-074 Me pretreatment showed the same inhibitory effect on berberine-induced cathepsin-B and AIF release (Figure 6B). Furthermore, CA-074 Me decreased cell death and LDH release induced by berberine in IMCE (Figure 6C–D) and in HT-29 (Figure S5B and S5C) cells. These results indicate that berberine-induced cathepsin B release mediates AIF activation and caspase-independent cell death in colon tumor cells.

### Berberine Activation of PARP Regulates AIF Activation and Cell Death in IMCE Cells

**Figure 7:**
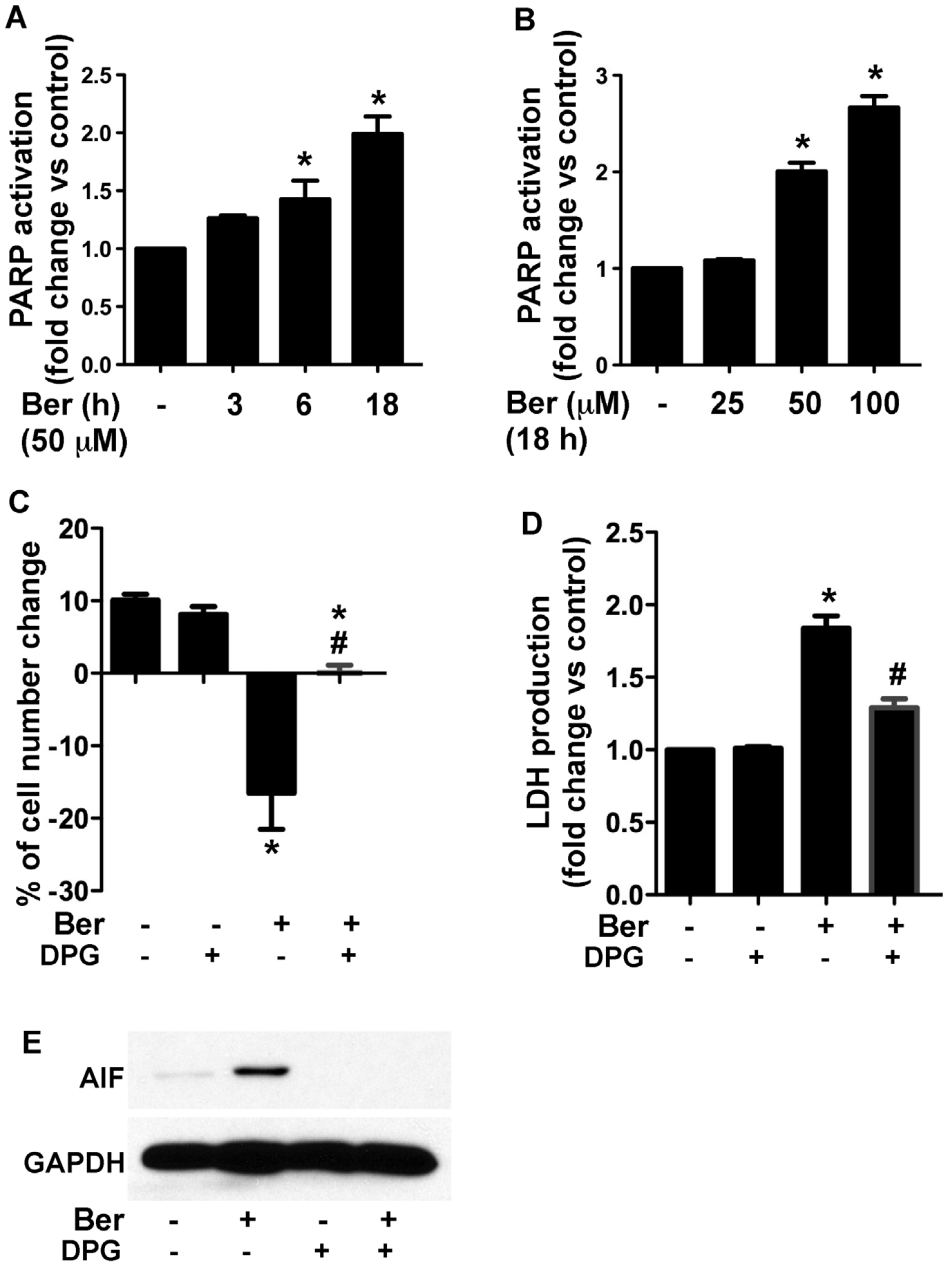
Berberine activates PARP in IMCE, which mediates berberine-mediated AIF activation and cell death. Cells were treated with berberine at 50 µM for the indicated times (A) or at the indicated concentrations for 18 h (B). PARP activity was measured using the HT 167 Universal Colorimetric PARP Assay Kit. PARP activation is expressed as a percentage of control in each experiment. Cells were treated with berberine at 50 µM for 18 h in the presence or absence of DPG (25 µM), a PARP activation inhibitor. Cell number change (C) and LDH release (D) were detected as described in Figure 2. The cytosolic fractions were prepared for Western blot analysis of AIF, as described in Figure 3 (E). *p<0.01 compared to the control group, #*p<*0.01 compared to the berberine treated group.

Studies have shown that PARP activation is another target for oxidative stress for stimulating AIF activation [21]. We tested whether PARP activation was involved in the berberine-induced mitochondrial AIF release in IMCE cells. Berberine activated PARP in IMCE cells in a time- and concentration-dependent manner (Figure 7A–B). The PARP activation inhibitor, DPG, prevented berberine-induced cell death (Figure 7C) and LDH release (Figure 7D) in IMEC and HT-29 (Figure S5B and S5C) cells. Moreover, DPG inhibited berberine-induced AIF release into the cytosol in IMCE cells (Figure 7E). Thus, activation of PARP may serve a mechanism by which berberine induces caspase-independent cell death through activation of AIF in colon tumor cells.

## Discussion

Considerable attention has been given to the identification of naturally occurring chemopreventive or chemotherapeutic reagents capable of inhibiting or reversing the tumor development process, including colon tumorigenesis. The evaluation of ancient herbal products that have been widely used for other diseases may provide insight into developing these herbs into new therapies for cancer treatment. Berberine, which has been used to treat bacteria-associated diarrhea, intestinal parasitic infections, and ocular trachoma infections for several decades, represents one of these herbs. In the present study, we showed that berberine decreased colon tumor colony formation and induced caspase-independent cell death in the IMCE colon tumor cell line. We further showed that the mechanism underlying berberine-induced cell death was through ROS-production-dependent AIF activation. Furthermore, two targets of ROS production in cells, cathepsin B release from lysosomes and PARP activation, have been shown to regulate AIF activation by berberine. Notably, YAMC cells, a normal colon epithelial cell line, were not susceptible to berberine-induced cell death and ROS production. Therefore, these results suggest that berberine may prevent and/or treat colon tumors through inducing caspase-independent cell death, with potentially little, or no side effects on normal colon cells.

The effects of berberine on inhibiting growth of a broad number of tumor cell types have been reported through various mechanisms, including suppressing proliferation and stimulating caspase-dependent apoptosis, through regulating signaling pathways, such as p53, MAPK, and NF-κB [7], [8]. The reported evidence suggests that the mechanism of berberine’s anti-tumor effects is tumor cell line-specific. For example, berberine has been reported to induce caspase-dependent apoptosis and disrupt mitochondrial membrane potential to release cytochrome C and AIF, which were prevented by overexpression of Bcl-X_L_, a signal to preserve mitochondrial integrity, in a melanoma cell line SK-MLE-2. Furthermore, results from this paper suggested that MEK/ERK and B-Raf signaling pathways, but not ROS production, were involved in berberine-induced apoptosis [22]. In our studies, when IMCE cells were treated with berberine, there were no effects on p53, MAPK, or NF-κB pathways (data not shown). A novel finding from our studies is the activation of AIF by berberine leading to caspase-independent cell death and the link between berberine-induced ROS generation and AIF activation in colon tumor cells. These results reveal a new mechanism for berberine’s action that may be important for colon tumor prevention and treatment. However, it should be noted that this may not be the sole mechanism for berberine’s activity in colon tumor cells.

The clinical efficacy of chemotherapeutic drugs is largely dependent on their ability to trigger tumor cell death, and activation of apoptosis is one of the well-studied mechanisms involved in this process. However, increasing evidence suggests that there are forms of chemotherapy-induced cell death that are different from apoptosis or necrosis [23]. Thus, strategies targeting AIF-mediated caspase-independent cell death have been applied as a therapeutic treatment for cancers. For example, a plant drug from *Scutellaria barbatae* that stimulates AIF translocation from the mitochondria to the nucleus showed anti-tumor effects in breast cancer patients [24]. Since cathepsin release from lysosomes regulates the AIF-mediated cell death pathway and several members of the cathepsin family have been implicated in cancer progression and metastasis [25], [26], cathepsin is a potential target to modulate caspase-independent cell death for cancer treatment. For example, paclitaxel poliglumex, which triggers disruption of the lysosomal integrity followed by cathepsin B release and cell death, has shown efficacy for patients with advanced non-small cell lung carcinoma in a phase III clinical trial [27]. Based on these encouraging results from clinical trials, berberine may be considered for further studies as a promising candidate for colon tumor prevention and treatment through modulating the AIF pathway leading to caspase-independent cell death in colon tumor cells.

Although caspase-dependent apoptosis plays a role in embryologic development and pathogenesis of several diseases, evidence suggests that death stimuli trigger alternative types of cell death in these physiological and pathological processes, such as cell death without the involvement of caspase activation. For example, caspase inhibition by zVAD-fmk exacerbated TNF toxicity by enhancing oxidative stress and mitochondrial damage, resulting in kidney failure and death in mice, which suggested the pathophysiological relevance of caspase-independent, ROS-mediated pathways in response to lethal TNF-induced shock [28]. Although berberine has been shown to induce caspase-dependent apoptosis in tumor cells [7], [8], it is also possible that the same stimulus can trigger cell death through caspase-dependent and caspase-independent mechanisms in different cell lines. For example, FAS ligand induced cell death in Jurkat cells in the absence of caspase activity, but caspase activity was required for FAS ligand-induced cell death in other cells [29]. Our results in the current study showed that the capase inhibitor, zVAD-fmk, suppressed cell death induced by TNF and cycloheximide, but not berberine, which is consistent with the result that TNF and cycloheximide, but not berberine stimulated caspase activation. These findings support our conclusion that berberine induces caspase-independent cell death. However, several possibilities should be noted, such as inefficiency of zVAD-fmk inhibition and the cytotoxicity of this inhibitor, which can itself induce cell death. zVAD-fmk alone did not stimulate cell death in ether IMCE or YAMC cells, and while it may have the potential to increase berberine’s cytotoxicity, we observed no such effect. In addition, the question of whether caspase-independent cell death is the sole method for cell death in berberine-treated tumor cells remains to be investigated. It is possible that berberine-induced caspase-independent cell death may trigger apoptosis in the later stage of berberine treatment.

ROS generation is implicated in the pathogenesis of many chronic diseases, including cancer, and therefore, the potential side effects of berberine-mediated ROS production should not be ignored. One interesting finding from our studies is that normal colon epithelial cells are less sensitive to berberine-induced cell death, compared to colon tumor cells. In YAMC cells, berberine did not induce ROS production (Figure 4), cell death (Figure 2A–B) or LDH release, whereas these treatment conditions induced cytotoxicity by ROS production in tumor cells. Berberine exerted similar cytotoxic effects on prostate cancer cells [20] and inhibited hepatoma cell invasion [30] through ROS production, but not in normal prostate [20] and liver cells [30]. These results indicate that normal cells, including colon epithelial cells, are resistant to berberine-induced oxidative stress. We have performed further experiments to investigate the mechanisms by which normal colon epithelial cells are less sensitive to berberine, and found that that absorption of berberine by YAMC is lower than that by IMCE cells (Figure S6). Thus, colon tumor cells are potentially specific targets of berberine.

In conclusion, the present study provides novel insight into the mechanism of berberine-induced caspase-independent cell death in both human and mouse colon tumor cells. These results will serve as the basis for *in vivo* studies to investigate the effects of berberine on colon tumor cancer development, which may make it a promising new relative selective therapeutic approach for colorectal cancer.

## Materials and Methods

### Cell Culture

YAMC is a conditionally immortalized murine colon epithelial cell line isolated from the mouse harboring thermolabile mutation (tsA58) under the control of an interferon (IFN)-γ-inducible H-2K^b^ promoter and a temperature-sensitive simian virus 40 large T antigen (Immotomouse) [31]. The functional expression of the SV40 large T antigen is induced by culturing the cells *in vitro* in medium containing IFN γ at a temperature permissive for function of the tsA58 mutation (33°C). Expression of this gene is required for YAMC cell proliferation. YAMC cells die when the temperature is raised to the non-permissive temperature (37°C) or when the cells are cultured without IFN-γ for three passages. The epithelial nature of YAMC cells has been characterized by expression of keratin 18, a marker of epithelial cells. Although the cells are morphologically primitive with no morphological evidence of differentiation such as formation of a brush border even when cultured at the non-permissive temperature, the cultured cells retain some properties of the intestinal tissue of origin, such as the presence of brush border associated enzymes, alkaline phosphatase, dipeptidyl peptidase IV, and sucrase.

IMCE cell line was generated from the colonic epithelium of F1 Immorto-*Apc* ^min/+^ mouse hybrid [32]. Thus, IMCE cells carry both the mutant *Apc* ^min^ gene and a temperature-sensitive mutant of the SV40 large T gene. YAMC and IMCE cell lines were provided by Dr. Robert Whitehead at Vanderbilt University, Nashville, TN.

YAMC and IMCE cells were maintained in RPMI 1640 medium supplemented with 5% fetal bovine serum (FBS), 5 U/ml of murine IFN-γ, 100 U/ml penicillin and streptomycin, 5 µg/ml insulin, 5 µg/ml transferrin, 5 ng/ml selenous acid on collagen coated plates at 33°C (permissive condition) with 5% CO_2_. HT-29 cells isolated from human colorectal adenocarcinoma (ATCC, HTB-38™) were grown in DMEM medium supplemented with 10% FBS at 37°C.

For the berberine treatment in the presence or absence of inhibitors, YAMC and IMCE cells were maintained in serum-starved RPMI 1640 medium containing 0.5% FBS and 100 U/ml penicillin and streptomycin (no IFN-γ) at 37°C (non-permissive condition), and HT 29 cells were cultured in serum-starved DMEM medium containing 0.5% FBS at 37°C.

### Transient Transfection of siRNA

IMCE cells were transiently transfected with either 20 nM non-targeting siRNA or 20 nM mouse AIF siRNA (Cell Signaling Technology) at 80% confluence using Lipofectamine 2000 (Invitrogen Corporation), according to the manufacturer’s instructions. After 24 h transfection at 33°C (permissive condition), cells were treated with berberine in serum-starved medium for 18 h at 37°C (non-permissive condition) for cell viability assay and LDH release assay. Cellular lysates were collected at the end of the experiment for Western blot analysis to determine the AIF expression level.

### Colony Formation Assay

IMCE cells with overexpression of v-Ha-*ras* gene (IMCE*^ras^*) were used to detect colon formation, as described previously [33]. Briefly, cells were prepared in 0.35% agar in IMCE cell culture medium, and overlaid onto the basal agar’s layer (0.5% agar in IMCE cell culture medium). Cells were cultured at 33°C (permissive condition) with 5% CO_2_ for 14 days for growth of colonies. Prior to count colonies, cells were stained using the CellTiter®120 AQueousOne Solution Cell Proliferation Assay (Promega) according to the manufacturer’s instructions. Colonies of >50 µm were counted by an automated colony counter (Biologics, Inc., Gainesville, VA).

### Cell Viability Assay

5,000 cells/well were plated in 96-well plates and cultured overnight to reach 70% confluence at 33°C (permissive condition) for IMCE and YAMC cells and at 37°C for HT 29 cells in normal medium. Then cells were treated with berberine in the presence or absence of a cell permeable ROS scavenger, Tiron (4,5-dihydroxy-1,3-benzenedisulfonic acid, Santa Cruz Biotechnology), a cell permeable cathepsin B inhibitor, CA-074 Me (L-3-trans- (Propylcarbamoyl)oxirane-2-Carbonyl)-L-soleucyl-L-Proline Methyl Ester, Enzo, Life Sciences), caspase inhibitor, zVAD-fmk (BD Pharmingen™), a PARP inhibitor, 3,4-dihydro-5-[4-(1-piperidinyl)butoxy]-1(2H)-isoquinolinone (DPQ), (Sigma-Aldrich), or a cell permeable caspase inhibitor, Z-VAD-FMK (Promega), at 37°C in serum-starved medium for both IMCE and YAMC (non-permissive condition) and HT 29 cells. Cell viability was assessed using the CellTiter®120 AQueousOne Solution Cell Proliferation Assay (Promega) according to the manufacturer’s instructions. The cell number standard curve was generated to determine the cell number in each treated group.

### LDH Release Assay

Cells were cultured and treated as described in the cell viability assay above. Cell culture supernatants were collected and LDH release was measured using the CytoTox 96® Non-Radioactive Cytotoxicity Assay (Promega) according to the manufacturer’s instructions. Data were expressed as percentages of control values.

### Detection of Mitochondrial Membrane Potential (Δψ)

***Δ*** *ψ* was measured using the Flow Cytometry Mitochondrial Membrane Potential Detection kit (BD™ Mitoscreen) according to the manufacturer’s instructions. Briefly, after treatment, cells were resuspended in 0.5 mL of JC-1 solution and incubated at 37°C for 15 min. After rinsing, cells were analyzed by flow cytometry. A dot plot of red fluorescence (living cells with intact ***Δ*** *ψ*) versus green fluorescence (cells with lost ***Δ*** *ψ*) was recorded. Data were expressed as percentage of cells with lost ***Δ*** *ψ*.

### Detection of Intracellular ROS Level

ROS production was detected using 2′,7′-dichlorofluorescein diacetate (DCFH-DA) (Molecular Probe), according to the manufacturer’s instructions. After treatment, cells were incubated with 10 µM of DCFH-DA dye in PBS for 15 min and washed with PBS 3 times. Fluorescence intensity of cells was analyzed using flow cytometry. Data were expressed as percentages of control values (DCF fluorescence in untreated cells).

### PARP Activation Assay

PARP activity in cells was detected using the Universal Colorimetric PARP Assay Kit (Trevigen, Inc., Gaithersburg, MD) according to the manufacturer’s instructions. Data were expressed as percentages of control values.

### Preparation of Cytosolic, Nuclear and Mitochondrial Fractions

Cells were scrapped in buffer-A containing 10 mmol/L Tris-HCl (pH 8), 1.5 mmol/L MgCl_2_, 1 mmol/L EDTA, 1 mmol/L 1,1,1-trichloro-2,2-bis(p-chlorophenyl)ethane; dichlorodiphenyltrichloroethane (DDT), 1 mmol/L phenylmethylsulfonylfluoride, 5 µg/mL pepstatin-A, 1 µg/mL leupeptin, and 2 µg/mL aprotinin. After centrifugation at 300×g for 10 min, the supernatant was saved as the cytosolic fraction. The cell pellet was resuspended in buffer-A plus 0.1% Triton X-100, and incubated on ice for 10 min. After centrifugation at 12,000×g for 10 min, the supernatant was saved as the mitochondrial fraction. The pellet was resuspended in buffer-B containing 20 mmol/L Tris-HCl (pH 8), 420 mmol/L NaCl, 1.5 mmol/L MgCl_2_, 1 mmol/L EDTA, 1 mmol/L DDT, 25% glycerol, 1 mmol/L phenylmethylsulfonyl-fluoride, 5 µg/mL pepstatin-A, 1 µg/mL leupeptin, and 2 µg/mL aprotinin, and agitated for 30 min at 4°C. The nuclear fraction was collected after centrifugation at 20,200×g for 10 min. Protein concentration was determined using a BCA protein assay kit (Pierce Thermo, Scientific). GAPDH (cytosolic marker), Ki-67 (nuclear marker), and COX IV (mitochondrial marker) were used to determine the purity of the fractions.

### Western Blot Analysis

For preparing total cellular proteins, cell monolayers were rinsed twice with cold PBS and then scraped into cell lysis buffer (50 mM Tris-HCl (pH 7.4), 120 mM NaCl, 1% NP-40) with protease and phosphatase 1 and 2 inhibitor cocktails (Sigma-Aldrich). The scraped suspensions were centrifuged (14,000×g for 10 min) at 4°C, and the protein concentration was determined using a BCA protein assay kit (Pierce Thermo, Scientific).

Equal amounts of total and fractionated proteins were mixed with Laemmli sample buffer and separated by SDS-polyacrylamide gel electrophoresis for Western blot analysis using antibodies against PARP (which identifies both un-cleaved (116 kDa) and cleaved PARP, Cell Signaling), cleaved caspase 3 (Cell Signaling), cleaved caspase 6 (Cell Signaling), cleaved caspase 9 (Cell Signaling), cleaved caspase12 (Cell Signaling), AIF (Cell Signaling), Cathepsin-B (Milipore), Ki-67 (Novocastra), GAPDH (Cell Signaling), COXIV (Cell Signaling), and β-actin (Santa Cruz Biotechnology).

### Immunostaining

After treatment, cells were washed with PBS and fixed using 4% paraformaldehyde for 15 min at room temperature. Cells were permebilized using 2% Triton X-100 for 5 min in PBS at room temperature and blocked in 3% bovine serum albumin in PBS containing 1% Triton X-100 for 1 h at room temperature. Slides were then incubated with anti-AIF antibody (Santa Cruz Biotechnology) or anti-cathepsin B (Ab-1) monoclonal antibody (Calbiochem) over night at 4°C and a FITC-labeled goat anti-rabbit IgG (Zymed) antibody at room temperature for 1 h. Slides were mounted using Vectashield™ Mounting Medium containing DAPI and observed using fluorescence microscopy. FITC and DAPI images were taken from the same field.

## Supporting Information

Figure S1 **Berberine induces cell death and LDH release in HT-29 cells.** HT-29 cells were cultured in serum-starved medium at 37°C for 24 h (A and B) or 36 h (C and D) with or without berberine (50 µM) treatment for indicated times from the end of experiment. Cell viability (A and C) and LDH release (B and D) were detected as described in Figure 2. Data represent 3 independent experiments, each performed in triplicate. *p<0.01 compared to the control group.
(TIF)

Figure S2 **High concentration of berberine induces LDH release, but not cell loss, in YAMC cells.** YAMC cells were treated with berberine in serum-starved medium at 37°C for 24 h at the indicated concentrations. Cell viability (A) and LDH release (B) were detected as described in Figure 2. Data represent 3 independent experiments, each performed in triplicate. *p<0.01 compared to the control group.
(TIF)

Figure S3 **Berberine-induced cell death in IMEC cells was detected by Annexin V and propidium iodide (PI) staining.** IMEC ells were treated with berberine (50 µM) in serum-starved medium at 37°C for 24 h. Then cells were dissociated using accutase and double stained with Annxin V-FITC and PI. The percentage of cells positive for Annexin V and PI was detected by flow cytometry. Viable cells have low Annexin V-FITC and low PI staining, early apoptotic cells have high Annexin V-FITC and low PI staining, late apoptotic cells and caspase-independent cell death have high Annexin V-FITC and high PI staining, necrotic cells have low Annexin V-FITC and high PI staining. The populations with high Annexin V (Annexin V+) and low PI (PI-) and Annexin V+PI+ are shown. Data represent 3 independent experiments, each performed in triplicate. *p<0.01 compared to the control group.
(TIF)

Figure S4 **Tiron inhibits berberine-induced ROS production in IMEC cells.** Cells were treated with berberine (50 µM) in serum-starved medium at 37°C for 18 h, as described in Figure 2, in the presence or absence of a ROS scavenger, Tiron (5 µM). Intracellular ROS levels were detected using DHE probe and the amount of fluorescence was measured using flow cytometry. The fold change of the amount of fluorescence was calculated by comparing that in the treated groups to the control group. Data represent 3 independent experiments, each performed in triplicate. *p<0.01 compared to the control group, #*p<*0.05 compared to the berberine only treated group.
(TIF)

Figure S5 **Berberine stimulates AIF release in IMCE, which is required for berberine-induced cell death in HT29 cells.** Cells were treated with berberine at 75 µM in serum-starved medium at 37°C for 24 h, as described in Figure 2. Cytoplasmic, nuclear, and mitochondrial fractions were prepared for Western blot analysis using anti-AIF, anti-GAPDH (cytosolic marker), anti-COX IV (mitochondrial marker) and anti-Ki-67 (nuclear marker) antibodies (A). Cells were treated with berberine at 75 µM for 24 h in the presence or absence of a ROS scavenger, Tiron (5 µM), a cathepsin B inhibitor, CA-074 Me (100 nM), or a PARP activation inhibitor, DPG (25 µM). Cell number change (B) and LDH release (C) were detected as described in Figure 2. Data represent 3 independent experiments, each performed in triplicate. *p<0.01 compared to the control group, #*p*<0.01 compared to the berberine only treated group.
(TIF)

Figure S6 **Absorption of berberine by IMEC and YAMC cells.** Cells were treated with berberine for 18 h at indicated concentrations (A) or at 50 µM for indicated times (B), as described in Figure 2. Cells were homogenized in PBS and the cellular soluble protein concentration was determined. Berberine content in the soluble fraction was determined by positive ionization LC-MS/MS using canadine as an internal standard. Data represent 3 independent experiments, each performed in triplicate. *p<0.01 compared to the control group, #*p*<0.01 compared to YAMC cells with same concentration (A) or time (B) of berberine treatment.
(TIF)
