# Supplementary figures and images for: Berberine Induces Caspase-Independent Cell Death in Colon Tumor Cells through Activation of Apoptosis-Inducing Factor
Source: PLoS One. 2012 May 4;7(5):e36418. doi: 10.1371/journal.pone.0036418 (PMC3344856; doi:10.1371/journal.pone.0036418)

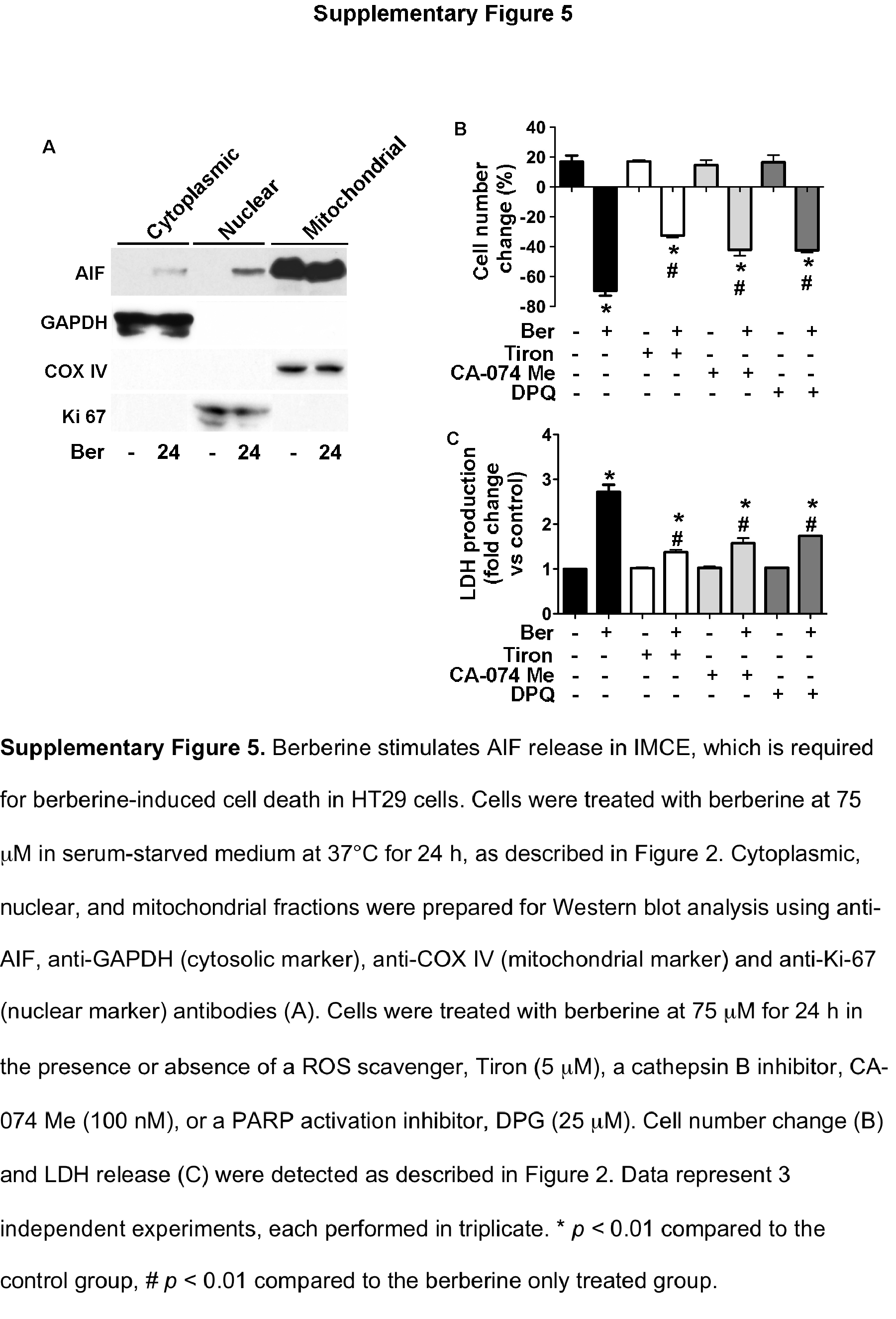

Supplement: Figure S5 — Berberine stimulates AIF release in IMCE, which is required for berberine-induced cell death in HT29 cells. Cells were treated with berberine at 75 µM in serum-starved medium at 37°C for 24 h, as described in Figure 2. Cytoplasmic, nuclear, and mitochondrial fractions were prepared for Western blot analysis using anti-AIF, anti-GAPDH (cytosolic marker), anti-COX IV (mitochondrial marker) and anti-Ki-67 (nuclear marker) antibodies (A). Cells were treated with berberine at 75 µM for 24 h in the presence or absence of a ROS scavenger, Tiron (5 µM), a cathepsin B inhibitor, CA-074 Me (100 nM), or a PARP activation inhibitor, DPG (25 µM). Cell number change (B) and LDH release (C) were detected as described in Figure 2. Data represent 3 independent experiments, each performed in triplicate. *p<0.01 compared to the control group, #p<0.01 compared to the berberine only treated group. (TIF) [file pone.0036418.s005.tif]

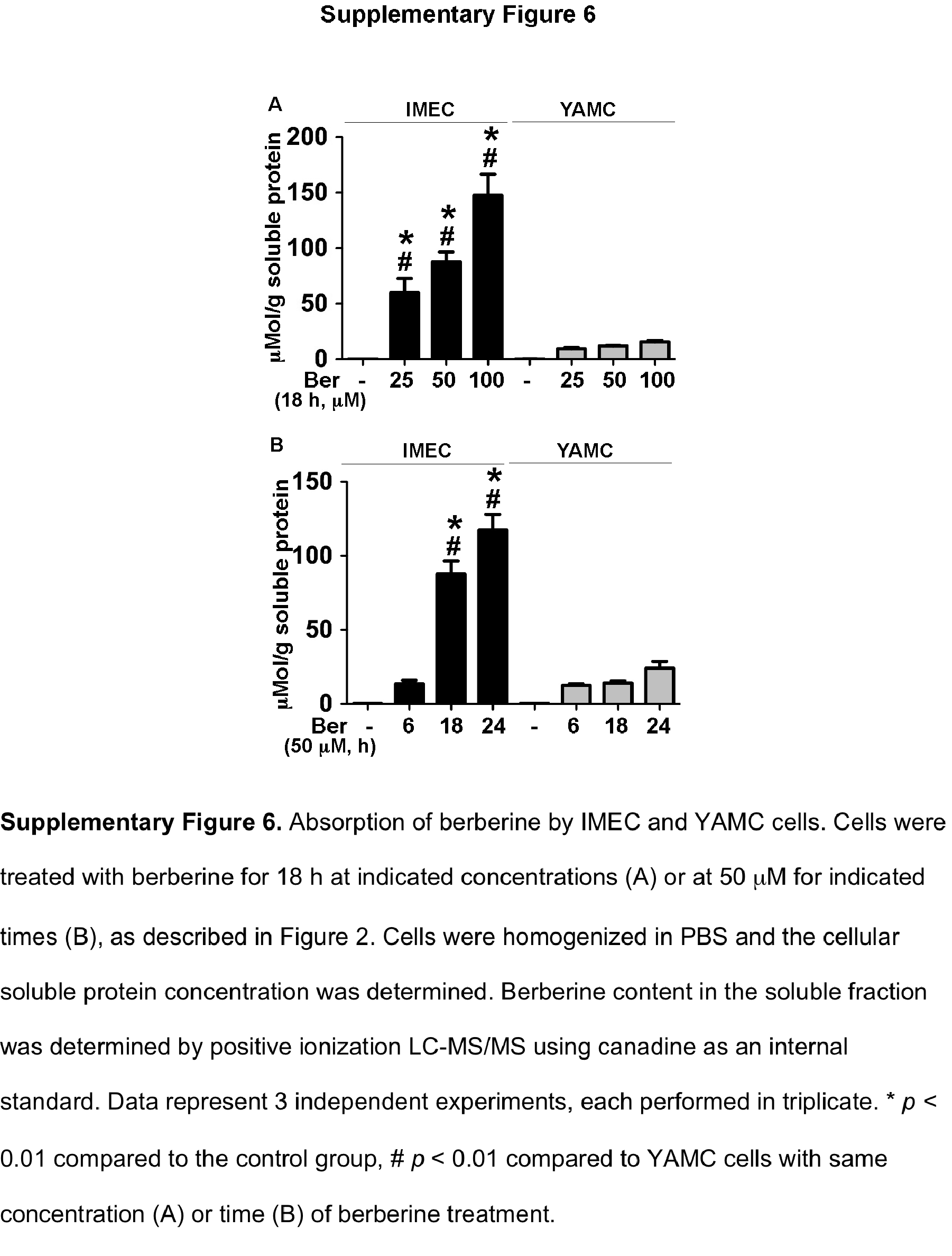

Supplement: Figure S6 — Absorption of berberine by IMEC and YAMC cells. Cells were treated with berberine for 18 h at indicated concentrations (A) or at 50 µM for indicated times (B), as described in Figure 2. Cells were homogenized in PBS and the cellular soluble protein concentration was determined. Berberine content in the soluble fraction was determined by positive ionization LC-MS/MS using canadine as an internal standard. Data represent 3 independent experiments, each performed in triplicate. *p<0.01 compared to the control group, #p<0.01 compared to YAMC cells with same concentration (A) or time (B) of berberine treatment. (TIF) [file pone.0036418.s006.tif]
